# State Registration and Its Values

**Published:** 1920-09-04

**Authors:** 


### THE MATRONS' AND SISTERS' DEPARTMENT.

### STATE REGISTRATION AND ITS VALUES.

### II. Reasons for Previous Opposition.

The value of a State Register of Nurses cannot be properly appreciated unless its limitations be understood. It ought to give valuable protection both to the public and to nurses themselves, but this it can only do if it be applied with discretion to ratify principles which have already been accepted. It was a clear comprehension of existing impediments to the smooth working of registration which set so many authorities against it in days gone by.

The first point which led to opposition was the danger that registration might be held by the public to afford a testimonial to character. It is inevitable that many nurses of very indifferent character will succeed in securing entrance to the Register. Or, to put it in another way, it is tolerably certain that not all the women whose good qualities and training suffice to place them on the Register will continue to walk in the narrow way. They may grow lazy and careless. They may take to drink or drugs. They may develop an unrestrained or cruel temper. In a hundred different ways they may deteriorate and become unsuited to attend upon the sick. But because they are "registered nurses" they may be able to command respect and procure employment. If they are convicted in a court of law their names can be struck off the Register, but hardly for any other reason. Everyone knows how exceedingly difficult it is to procure evidence of intemperance which will satisfy legal opinion, and "professional incompetence" is almost equally hard to prove. Thus, said the opponents of registration, the Register will be misunderstood, and will prove a source of danger to the public.

The next drawback foreseen was the wholesale admission of the *bona-fide* nurse to the Register, without which proviso it was well known the House of Commons would never pass a Registration Bill. Twenty or more years ago, when the battles over Registration began, the *bona-fide* nurse was a very different product from the *bona-fide* nurse of the present day. She was often simply and solely a person who had been employed in sick nursing in private families or at nursing homes, without a shadow of training. She was what would now be called a sick-room attendant. The authorities who had in charge to safeguard the interests of their nurses objected very strongly indeed to a measure which would have completely confused the issue as between trained nurses and the untrained, for the space of a whole generation. In their judgment the criterion of training should be the certificate. The nurse who possessed the certificate of a reputable training-school could always find employment. It was unjust to her to create an easily obtained professional hall-mark called Registration, and support it with the authority of the State. It meant unfair competition.

A third objection made to registration in days gone by was the danger which was foreseen of its crystallising a standard inferior to that which many matrons with high educational aims were then struggling to secure. Certain hospitals had instituted a three-year course, one or two had a four-year course; but a large number of hospitals were still giving a two-year course. Which would be selected by the State if it were called upon to set up a Registration authority? Of course it was well known that the principal advocates of Registration had declared in favour of a three-year course, but when once the aid of the Government was invoked powerful interests would make themselves felt, and the issue was recognised as very doubtful. The example of America, where a two-year course had been fixed by law in many States, was not encouraging. If nurses were to be assured of State Registration under a two-year course, the fate of progressive nursing education would be sealed.

For these reasons there was no denying that the forces opposed to Registration in the nursing world were far stronger than those in favour of it. Nurses were not interested in it as a body. They were not prepared for it. Had it been forced upon the profession by an insignificant minority, it was practically certain that the great body of well-trained and certificated nurses in the kingdom would have stood aloof from it. Overloaded with an immense preponderance of untrained, half-trained, or in some way doubtful nurses, it would have been supported merely by a few training-schools, or by a fraction of the trained nurses of the kingdom. It would have hopelessly divided the profession. After a short time there would have been (*a*) registered nurses without training; (*b*) registered and certificated nurses; (*c*) unregistered and untrained nurses; (*d*) certificated nurses despising Registration. And it was perfectly evident that it was among the last-named category that some of the best nurses would be found. Would not the State Register have been worse than useless under these circumstances?

In order to understand the previous opposition of some of the present members of the College of Nursing to Registration, this complicated position of affairs not many years ago must be taken into consideration.

*(To be continued.)*

